# Ex Vivo and In Vivo Analysis of a Novel Porcine Aortic Patch for Vascular Reconstruction

**DOI:** 10.3390/ijms22147623

**Published:** 2021-07-16

**Authors:** Ignacio Stöwe, Jens Pissarek, Pia Moosmann, Annica Pröhl, Sven Pantermehl, James Bielenstein, Milena Radenkovic, Ole Jung, Stevo Najman, Said Alkildani, Mike Barbeck

**Affiliations:** 1 Helios Clinic Emil-von-Behring, Department of Vascular and Endovascular Surgery, 14165 Berlin, Germany; Ignacio.stoewe@gmx.net; 2 Clinic and Policlinic for Dermatology and Venereology, University Medical Center Rostock, 18057 Rostock, Germany; Sven.pantermehl@med.uni-rostock.de (S.P.); james.bielen@gmail.com (J.B.); ole.tiberius.jung@gmail.com (O.J.); 3 biotrics bioimplants AG, 12109 Berlin, Germany; jens.pissarek@biotrics.com (J.P.); pia.moosmann@biotrics.com (P.M.); 4 BerlinAnalytix GmbH, 12109 Berlin, Germany; annica.proehl@berlinanalytix.com (A.P.); Said.alkildani@berlinanalytix.com (S.A.); 5 Scientific Research Center for Biomedicine, Department for Cell and Tissue Engineering, Faculty of Medicine, University of Niš, 18000 Niš, Serbia; Milena1390nis@gmail.com (M.R.); stevo.najman@gmail.com (S.N.); 6 Department of Biology and Human Genetics, Faculty of Medicine, University of Niš, 18000 Niš, Serbia; 7 Department of Ceramic Materials, Chair of Advanced Ceramic Materials, Institute for Materials Science and Technologies, Technical University Berlin, 10623 Berlin, Germany

**Keywords:** vascular reconstruction, vascular grafts, xenografts, porcine aorta, bovine pericardium, macrophages, inflammation

## Abstract

(1) Background: The aim of the present study was the biocompatibility analysis of a novel xenogeneic vascular graft material (PAP) based on native collagen won from porcine aorta using the subcutaneous implantation model up to 120 days post implantationem. As a control, an already commercially available collagen-based vessel graft (XenoSure^®^) based on bovine pericardium was used. Another focus was to analyze the (ultra-) structure and the purification effort. (2) Methods: Established methodologies such as the histological material analysis and the conduct of the subcutaneous implantation model in Wistar rats were applied. Moreover, established methods combining histological, immunohistochemical, and histomorphometrical procedures were applied to analyze the tissue reactions to the vessel graft materials, including the induction of pro- and anti-inflammatory macrophages to test the immune response. (3) Results: The results showed that the PAP implants induced a special cellular infiltration and host tissue integration based on its three different parts based on the different layers of the donor tissue. Thereby, these material parts induced a vascularization pattern that branches to all parts of the graft and altogether a balanced immune tissue reaction in contrast to the control material. (4) Conclusions: PAP implants seemed to be advantageous in many aspects: (i) cellular infiltration and host tissue integration, (ii) vascularization pattern that branches to all parts of the graft, and (iii) balanced immune tissue reaction that can result in less scar tissue and enhanced integrative healing patterns. Moreover, the unique trans-implant vascularization can provide unprecedented anti-infection properties that can avoid material-related bacterial infections.

## 1. Introduction

Diseases of the vascular system affecting damages of both the coronary arteries and peripheral blood vessels are the primary causes of death in the world [1]. The treatment of vascular diseases often involves, besides the endovascular branch, open procedures such as cardiac and peripheral bypass surgeries in which the damaged segment of blood vessels is replaced by appropriate vascular grafts [2,3,4,5]. Up to date the replacement materials can be divided into five different classes: (a) autogenous grafts [6,7], (b) allografts [8,9], (c) alloplastic or synthetic grafts [10,11,12], (d) processed xenografts [13,14,15,16], and biohybrid grafts [17,18]. Despite constant efforts to find a suitable material for bypass grafting, autogenous blood vessel transplants are still the gold standard and are mainly based on veins that are not crucial for the patient (for example, the great saphenous vein or small saphenous vein) [19]. However, malignant intimal hyperplasia with reduction in the lumen and the consequent stenosis or occlusion still remains a major problem when using autogenous veins as a bypass or as a patching material [20,21]. Moreover, restrictions such as limited usable autologous veins due to other related co-diseases such as varicose degeneration, thrombosis, intramural calcification or previous vessel replacement surgeries, or the inappropriate size of the transplant (compliance mismatch) led to the necessity of the application of replacement materials [22,23].

Broad research was conducted to develop structurally modified biological grafts with the goal of creating a durable non-immunogenic graft. Substantial efforts have been made toward the development of xenogeneic vascular replacement materials [24]. In this context, acellular extracellular matrix (ECM) scaffolds derived from xenogeneic vascular tissues have manifoldly been supposed to have the potential to overcome the aforementioned challenges [25,26]. Furthermore, they offer increased patency, unlimited availability, and sufficient length to serve as “off-the-shelf” vascular grafts. These biodegradable materials also provide increased resistance to infections and, thanks to their metabolic activity, can be treated with antibiotics [27,28]. Moreover, there is growing evidence that biological substitute materials are superior to synthetic materials, regardless of the antimicrobial additives, in view of the risk of reinfection [29]. In this context, the vascularization of the vascular graft and its implant bed is crucial in terms of implant integration and especially of resisting infections [30,31]. With sufficient vascularization, the implant is diffused not only with nutrients but also with (immune) cells and antibodies of the innate and humoral immunities [30,31]. Additionally, administered antibiotics as treatment or management of vascular graft infection are hypothesized to be better diffused within the implantation bed [32].

One of the main limitations includes the necessity for the removal of all potential immunologic components such as cells and proteins that may induce epitope-related graft rejections or (severe) foreign body reactions within the recipient organism. In this context, the three main conditions for scaffold failure have been identified: (a) an increased pro-inflammatory immune response, (b) altered mechanical properties, and (c) improper recellularization and vascularization [33]. The overall goal of every decellularization process is the best possible preservation of the extracellular matrix, i.e., the collagen network and structure, while the immunologic components should be removed as far as possible. Different pretreatment protocols were applied and showed promising results in the medium term [34]. Thus, a balance between decellularization and matrix-preservation but also the preservation of the mechanical properties is the final goal. As the basis of the present study, a novel xenogeneic vascular graft material based on native collagen won from porcine aorta was developed by adopting an already established protocol that was used for the purification of dental collagen-based biomaterials. These materials have already been shown to be adequately decellularized and not to elicit an exaggerated pro-inflammatory tissue reaction [35,36,37]. As a control, an already commercially available collagen-based vessel graft (XenoSure^®^, LeMaitre Vascular, Vaughan, ON, Canada) based on bovine pericardium was used. The aim of the present study was to analyze the (ultra-) structure and the purification effort based on previously described histological methods [38]. Furthermore, an established subcutaneous implantation model in Wistar rats was conducted to test the biocompatibility and the integration behavior of the novel graft material and the control material. Established methods combining histological, immunohistochemical, and histomorphometrical procedures were applied to analyze the tissue reactions to the vessel graft materials, including the induction of pro- and anti-inflammatory macrophages to test the immune response [38,39].

## 2. Results

### 2.1. Biomaterials

#### 2.1.1. Porcine Vessel Graft

**Figure 1:**
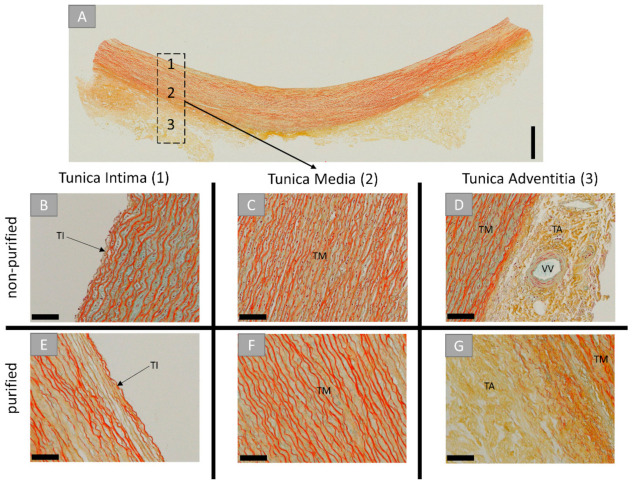
(**A**) Cross-sectional overview and detail microscopic images prior (**B**–**D**) and after decellularization (**E**–**G**) of the porcine aorta. TI: tunica intima, TM: tunica media, TA: tunica adventitia, VV: vasa vasorum. (Movat pentachrome stainings, (**A**) “total scan”, scalebar = 1 mm, (**B**–**F**) 20× magnifications, scale bars = 100 µm).

The histological analysis of the novel vessel graft, the porcine aorta patch (PAP), included the origin tissue prior to the decellularization process and the purified biomaterial (Figure 1). The analysis showed that the origin tissue material was consisting of three distinct layers or tunicae, which were also identifiable after purification (Figure 1A). The most internal one was the tunica intima, including an internal elastic lamina and endothelial cells (Figure 1B). The tunica media is the middle layer, and it contains smooth muscle cells that are interposed with elastic fibers (Figure 1C). The most outer layer, the tunica adventitia, contained connective tissue combining collagen and elastic fibers in concert with mainly fibroblasts (Figure 1D). Within this layer, vasa vasorum were found (Figure 1D). Post-purification, the extracellular structures of all tunicae remained unharmed (Figure 1E–G). No signs of cellular components or remnants were found, and only the acellular components of the tunicae were observable.

#### 2.1.2. Bovine Vessel Graft

**Figure 2:**
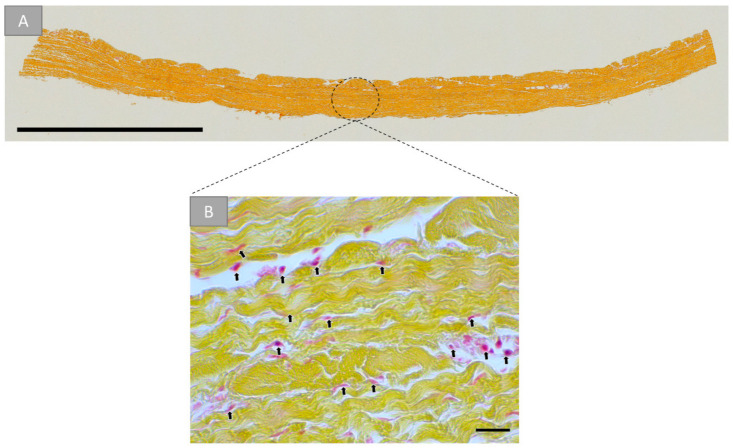
(**A**) Cross-sectional overview and detail microscopic image (**B**) of the purified bovine pericardial tissue. Arrows: remaining donor cells (Movat pentachrome stainings, (**A**) “total scan, scalebar = 2 mm, (**B**) 40× magnifications, scale bars = 20 µm).

The histological analysis of the commercially available bovine pericardial patch (BPP) is shown in (Figure 2). A detailed microscopic closeup allows the visualization of a predominantly collagen-based makeup of this tissue (Figure 2B). Cell debris was observed in the closeups (Figure 2B).

### 2.2. Results of the Cytocompatibility Analysis

#### 2.2.1. Histopathological Results

**Figure 3:**
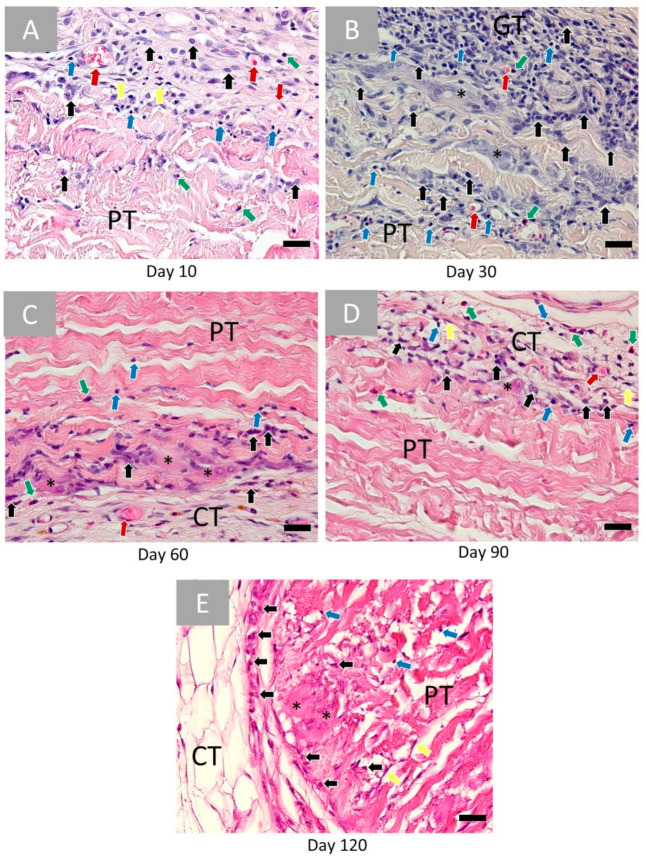
Histopathological images of the bovine pericardium-based vascular patch (PP) at (**A**) 10, (**B**) 30, (**C**) 60, (**D**) 90 and (**E**) 120 days post implantationem within the subcutaneous connective tissue (CT). Red arrows: vessels, black arrows: macrophages, blue arrows: lymphocytes, yellow arrows: fibroblasts, green arrows: eosinophils, and black asterisks: multinucleated giant cells (HE-stainings, 40× magnifications, scale bars = 20 µm).

The bovine pericardium patch (BPP) induced an initial tissue reaction at day 10 implantationem including a granulation tissue at both material surfaces (Figure 3A). It contained inflammatory cells such as granulocytes and mainly eosinophils, lymphocytes, fibroblasts, and macrophages (Figure 3A). Blood vessels were also visible within the granulation tissue (Figure 3A). At this early time point, cells invaded only the superficial material regions, while the material cores were free of cellular ingrowth.

At day 30 post implantationem, similarly composed granulation tissue was detectable at the material surfaces (Figure 3B). At this time point, mainly macrophages infiltrated practically one-third of the biomaterial, while the inner material cores were still free of cells (Figure 3B). Moreover, blood vessels and multinucleated giant cells (MNGCs) were visible at the material surfaces and within the infiltrated areas (Figure 3B).

At day 60 post implantationem, there is a “passivation” of the inflammatory tissue response (Figure 3C). Thus, the cell numbers were decreased, and mainly macrophages and MNGCs were found at the surface of the patch and within the superficial regions, while the material cores were cell-free (Figure 3C). Additionally, moderate numbers of blood vessels were observable within the connective tissue adjacent to the patch (Figure 3C).

A minor reactive tissue was still present at the surface of the patch at day 90 post implantationem that was comparable to the cellular composition at day 60 (Figure 3D).

At day 120 post implantationem, the surrounding reactive tissue showed a comparable composition as also found at days 60 and 90 (Figure 3E). Mostly macrophages and MNGCs were detectable at the surfaces of the patch (Figure 3E). Throughout all time points, the BPP patch, implanted subcutaneously, remained intact, and no hemorrhage nor necrosis was spotted.

**Figure 4:**
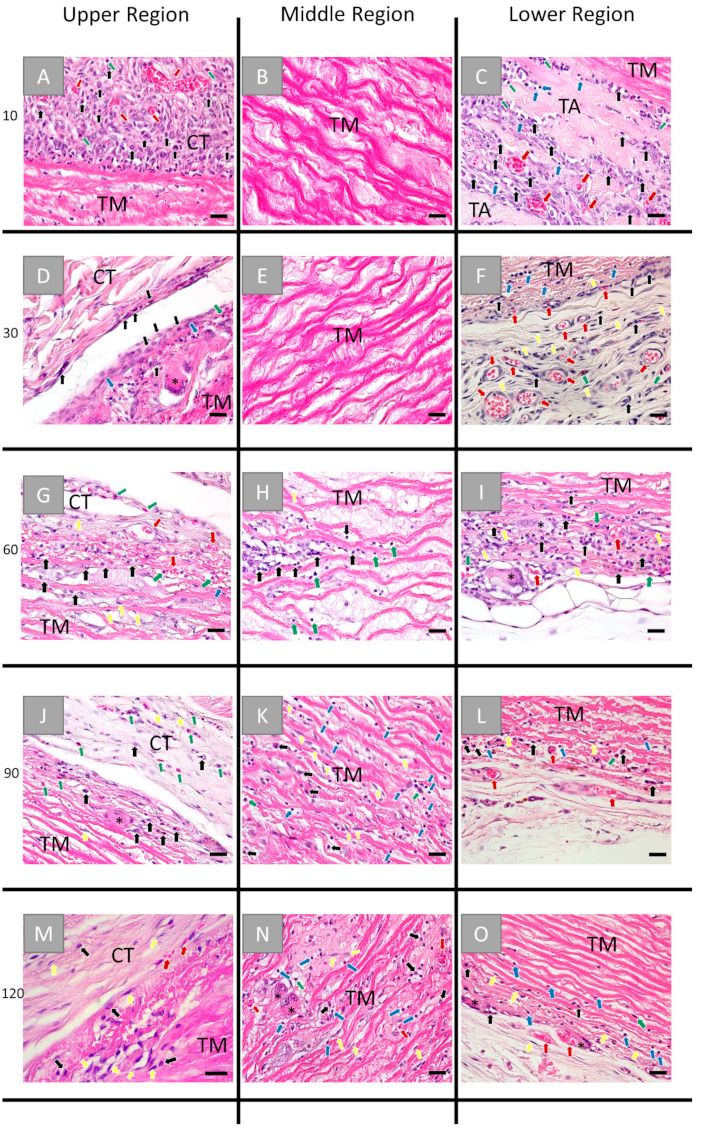
Histological images of the tissue reaction to the 3 different parts, i.e., the former tunica intima (left row), the tunica media (middle row), and the tunica adventitia (right row), of the porcine aorta patch after (**A**–**C**) 10, (**D**–**F**) 30, (**G**–**I**) 60, (**J**–**L**) 90, and (**M**–**O**) 120 days post implantationem. CT: connective tissue, TM: tunica media, TA: tunica adventitia, red arrows: vessels, black arrows: macrophages, blue arrows: lymphocytes, green arrows: granulocytes, yellow arrows: fibroblasts and black asterisks: multinucleated giant cells. (HE-stainings, 40× magnification, scale bars = 20 µm).

In contrast, the histopathological analysis in the group of the porcine aorta patch could be divided into three varying parts as every anatomical component or tunica (also described in Section 2.1.1) induced a different tissue response and underwent another integration behavior up to day 60 post implantationem (Figure 4).

At day 10 post implantationem, an inflammatory tissue reaction was seen at all outer surfaces of the patch, including macrophages, granulocytes, and mainly eosinophils, lymphocytes, fibroblasts, as well as some blood vessels (Figure 4A,C). At this time point, no cells infiltrated the inner core of the patch, i.e., the former tunica muscularis (Figure 4B). Interestingly the extracellular connective tissue components of the former tunica adventitia appeared fragmented and bundle-like and were surrounded by reactive tissue with visibly more blood vessels and above-described inflammatory cell types compared to the upper region, i.e., the former tunica intima (Figure 4C).

At day 30 post implantationem, the granulation tissue composed of granulocytes (i.e., mainly eosinophils), lymphocytes, and macrophages still surrounded the implant, while the inner core was free of cellular ingrowth (Figure 4D–F). At this time point, a few multinucleated giant cells (MNGCs) were found adhering to the surface of the implant representing the former tunica intima (Figure 4D). The tunica adventitia was most often completely degraded, and a highly vascularized reactive tissue, including granulocytes, lymphocytes, fibroblasts, and macrophages, was observed within this implant region (Figure 4F).

At day 60 post implantationem at both material surfaces (i.e., the former tunica intima and tunica adventitia), only a low manifestation of the previously described tissue reaction pattern was visible in a comparable pattern at both material surfaces, and the first cell invaded the inner material core (former tunica muscularis) at this time point (Figure 4G–I). Thereby, the tissue reaction to both material surfaces included mainly macrophages beside lower numbers of eosinophils and fibroblasts but also single lymphocytes (Figure 4G,I). In addition, single MNGCs were found in concert with the aforementioned cell types (Figure 4G,I). Blood vessels were visible within the adjacent connective tissue and the superficial layers at the former tunica intima and tunica adventitia (Figure 4G,I). At this timepoint, single cells have infiltrated the tunica media, which were mainly belonging to the macrophage and the granulocyte line, i.e., eosinophils (Figure 4H).

At day 90 post implantationem, the manifestation of the inflammatory tissue reaction was even lower compared to the former study time point (Figure 4J–L). Thus, only single cells, i.e., mainly macrophages and eosinophils beside single MNGCs, were found at both material surfaces (Figure 4J,L). The surrounding connective tissue contained a still higher number of blood vessels at this time point (Figure 4L). The tunica media has further infiltrated mainly macrophages, fibroblasts, and eosinophils (Figure 4K).

At day 120 post implantationem, the manifestation of the inflammatory tissue reaction was visibly lower compared to the former study time points (Figure 4M–O). Still, mainly macrophages, fibroblasts, and eosinophils, besides a few MNGCs, were involved in the tissue reactions to the former tunica intima and tunica adventitia (Figure 4M,O). The tunica media was highly infiltrated by eosinophils, lymphocytes, fibroblasts, and macrophages in concert with blood vessels and single MNGCs (Figure 4N).

#### 2.2.2. Analysis of the Immune Response

**Figure 5:**
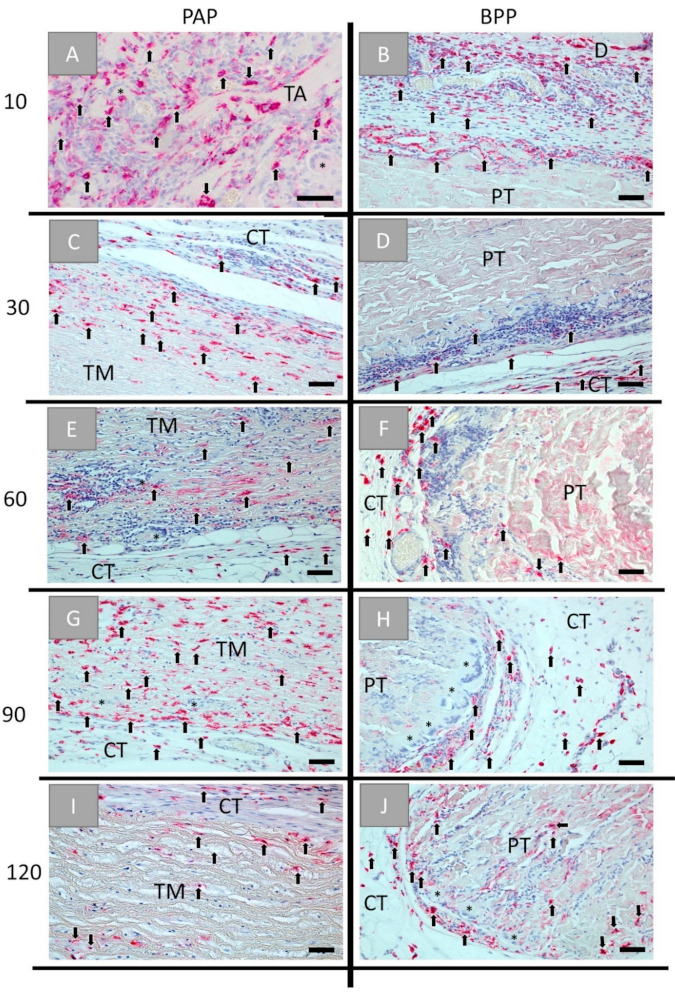
Histological images of the immunohistochemical detection of anti-inflammatory cells within the implantation beds of the porcine aorta patch (PAP, left row) and the bovine vessel patch (BPP, right row) at (**A**,**B**) day 10, (**C**,**D**) day 30, (**E**,**F**) day 60, (**G**,**H**) day 90, and (**I**,**J**) day 120 post implantationem within the subcutaneous connective tissue (CT). Black arrows: CD163-postive macrophages, black asterisks = CD163-negative MNGCs, TM: tunica media, PT: pericardial tissue (Immunohistochemical CD163-stainings, 20× magnifications, scale bars = 50 µm).

The detection of anti-inflammatory cells revealed that comparable numbers of CD163-positive macrophages were found within the implantation beds of both biomaterials over the whole time course of the study (Figure 5). Thereby, the porcine aorta patch was infiltrated early on by anti-inflammatory macrophages starting from day 60 post implantationem, while this invasion behavior was observed in the bovine pericardial patch starting with day 120 post implantationem (Figure 5E–J). The observed MNGCs did not show any expression of the CD163 marker molecule in both study groups (Figure 5).

**Figure 6:**
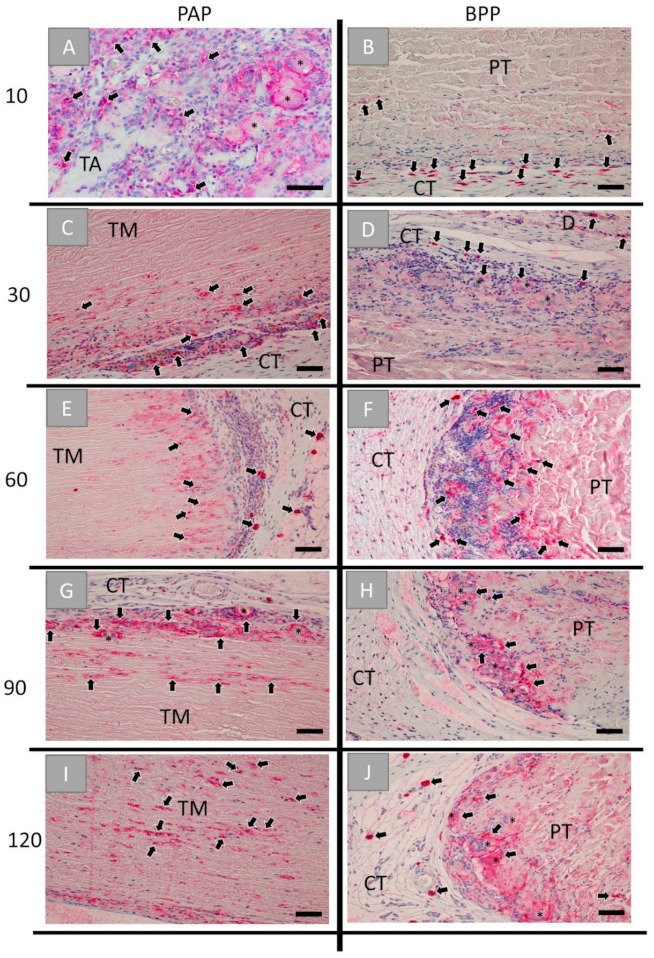
Histological images of the immunohistochemical detection of pro-inflammatory cells within the implantation beds of the porcine aorta patch (PAP, left row) and the bovine vessel patch (BPP, right row) at (**A**,**B**) day 10, (**C**,**D**) day 30, (**E**,**F**) day 60, (**G**,**H**) day 90 and (**I**,**J**) day 120 post implantationem within the subcutaneous connective tissue (CT). Black arrows: CD11c-postive macrophages, black asterisks = CD11c-positive MNGCs, TM: tunica media, PT: pericardial tissue, D: dermis (immunohistochemical CD11c-stainings, 20× magnifications, scale bars = 50 µm).

The detection of pro-inflammatory cells showed that CD11c-positive macrophages were mainly located onto the surfaces or the superficial regions of both biomaterials up to day 90 post implantationem (Figure 6A–H). Starting with day 120 post implantationem, pro-inflammatory cells started to invade both materials (Figure 6I,J). With the exception of day 10 and day 120 post implantationem, comparable numbers of pro-inflammatory cells were found in both study groups, while higher numbers of cells expressing the CD11c-molecule were found in the group of the bovine vessel patch at both aforementioned time points (Figure 6). Moreover, the MNGCs found within both study groups showed expression of the CD11c-molecule (Figure 6).

**Figure 7:**
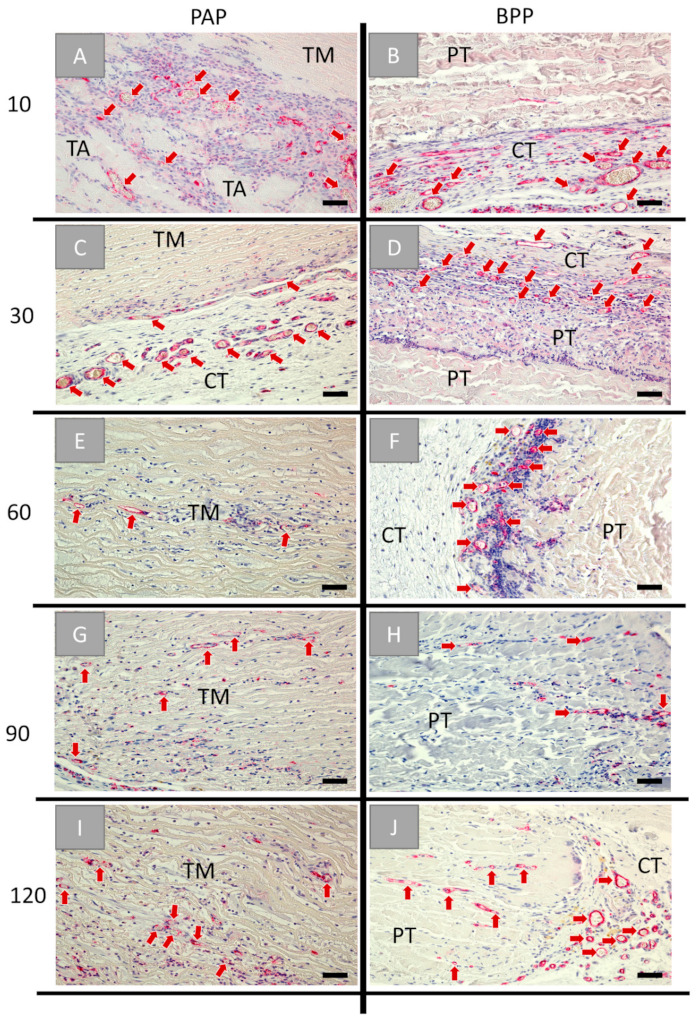
Histological images of the immunohistochemical CD31-detection within the implantation beds of the porcine aorta patch (PAP, left row) and the bovine vessel patch (BPP, right row) at (**A**,**B**) day 10, (**C**,**D**) day 30, (**E**,**F**) day 60, (**G**,**H**) day 90, and (**I**,**J**) day 120 post implantationem within the subcutaneous connective tissue (CT). Red arrows: blood vessels, TA: tunica adventitia, TM: tunica media, PT: pericardial tissue (Immunohistochemical CD31-stainings, 20× magnifications, scale bars = 50 µm).

The immunohistochemical vessel detection showed that the granulation tissue surrounding both biomaterials at day 10 post implantationem was accompanied with high vessel numbers, while the material bodies were free of vessels (Figure 7A,B). In the case of the porcine aorta patch, the vascularization was increased surrounding the tunica adventitia components (Figure 7A). At day 30 post implantationem, the vessels were also detectable within the adjacent connective tissue in both groups (Figure 7C,D). In the case of the porcine aorta patch, vascularization was mainly observed in the areas where the former tunica adventitia was located (Figure 7C). At day 60 post implantationem, vessels were infiltrating the former tunica media of the aorta patch, while in the group of the BPP patch vessels remained only in the periphery of the implant (Figure 7E,F). At day 90 post implantationem, more vessels were detected within the core of the tunica media of the porcine aorta patch, while vascularization remained in the periphery of the BPP patch (Figure 7G,H). At day 120 post implantationem, vascularization increased within the cores of the porcine vessel graft (Figure 7I), and vessels started to invade also the central regions of the bovine patches (Figure 7J).

### 2.3. Histomorphometrical Results

**Figure 8:**
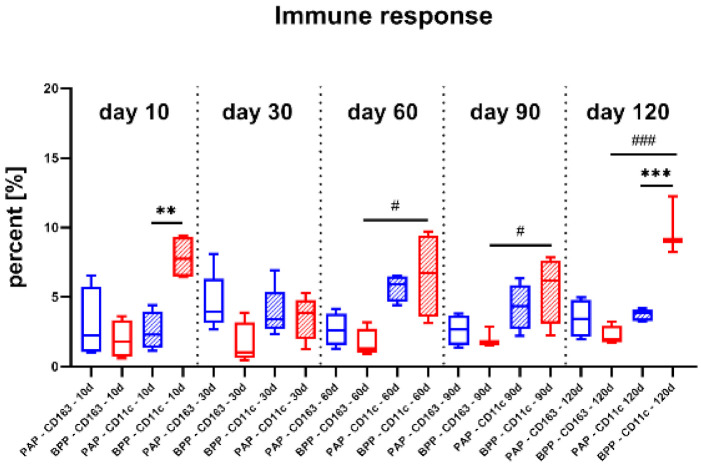
Results of the histomorphometrical analysis of the occurrence of pro- and anti-inflammatory cells (* = interindividual significances, # = intraindividual significances, # *p* < 0.05, ** = *p* < 0.01, ***/### *p* < 0.001). PAP: porcine aortic patch, BPP: bovine pericardial patch, blue: PAP-CD163, red: BPP-CD163, blue patterned: PAP-CD11c, red patterned: BPP-CD11c.

The histomorphometrical analysis of the occurrence of pro- and anti-inflammatory cells revealed that both materials induced comparable amounts of anti-inflammatory cells at day 10 post implantationem (Figure 8 and Table 1). Furthermore, the PAP graft induced comparably low numbers of pro-inflammatory cells, while the BPP graft induced pro-inflammatory cell amounts that were significantly higher than the values in the PAP group but also higher than the anti-inflammatory cell amounts in the same study group (##/** *p* < 0.01) (Figure 8).

At day 30 post implantationem, comparable amounts of pro- and anti-inflammatory cells were found in both study groups, while a tendency toward an anti-inflammatory cell response was detected in the PAP group (Figure 8 and Table 1).

At 60 and 90 days post implantationem, the percentages of anti-inflammatory cells were comparable without significant differences between both study groups (Figure 8 and Table 1). In both groups, higher amounts of pro-inflammatory cells were measured, while only in the BPP group, the amounts of pro-inflammatory cells were significantly higher (# *p* < 0.05) than the anti-inflammatory subtypes at both time points (Figure 8).

Histomorphometry at day 120 post implantationem showed percentages of anti-inflammatory cells in both study groups (Figure 8 and Table 1). However, the percentage of pro-inflammatory cells was significantly higher (*** *p* < 0.001) in the BPP group compared to the amounts in the PAP patch. In addition, significantly higher percentages of pro-inflammatory cells appeared (### *p* < 0.001) were measured in the BPP group compared to the percentages of anti-inflammatory cells (Figure 8).

## 3. Discussion

Although autografts are the gold standard grafts that were most likely used in the initial vascular reconstruction, this graft type is not ideal and, at times, not applicable due to donor site morbidity that limits their availability, size mismatches, and further complications [22,23]. Size mismatch leads to flow limitation and raises the chance for graft failure what can extend operative time or make a revision necessary and consequently increases the chance of infection [32]. Therefore, cryopreserved allogenic grafts and alloplastic grafts have been used as alternatives to autografts in vascular reconstruction [8,9,10,11,12]. Allografts require less operative time but come with the risks of occlusion and implant failure due to their compromised mechanical properties [32]. Synthetic grafts have been used since the 1950s, and although their high morbidity rates have decreased with their development, this graft type lacks bioactivity and matching of mechanical properties with the native tissue [30,40]. Their lack of bioactivity does not allow for optimal integration of the implant with the surrounding tissue nor for its vascularization [40,41]. This subsequently allows for the buildup of bacterial biofilm, which is an initiating factor of the antibiotic-resistant and delayed infection [30,42]. Additionally, compliance mismatching between the native vascular tissue and the synthetic graft has been reported to cause stenosis due to intimal hyperplasia [43].

Alternatively, xenografts can be used to overcome the complications that accompany the current vascular graft options [43,44]. Despite the fact that bovine pericardium is currently the most commonly used xenograft in vascular reconstruction, it is not as extensively investigated as PTFE and PE [45]. Moreover, even though bovine pericardium exhibited lower reinfection rates than PTFE and PE, clinical observations were reported only for short-term outcomes that are not enough to eliminate the possibility of a delayed infection, which has been reported recently [46]. Another limitation of bovine collagen is the IgE-mediated allergic reaction that is reported to be induced in up to 3.8% of the examined populations [47,48].

In this context, it is well known that infection of vascular grafts represents one of the most challenging complications in vascular reconstruction, increasing rates of limb amputations and, in many cases, increasing mortality [30]. Contamination of the graft and its adjacent tissue can furthermore lead to the disruption of anastomoses and following erosion of the implant. Infections can occur within the early (<4 months) or late (>4 months) post-implantation phase [30]. Samson et al. classified the degree of vascular graft infections upon which the treatment protocol is decided (Table 2) [49].

Classes I–II are for infections that are in the surrounding tissues and can be treated with either operative debridement, intravenous antibiotics, or negative pressure wound therapy [30,49]. However, classes III–V are often occurring in patients that have bacteremia and bleeding at the anastomosis site (Table 2). These classes also indicate the invasion of bacteria on the body of the graft, on the anastomosis, and within surrounding native tissue. Classes III–V often require the excision of the infected graft and the reimplantation of a new vascular graft.

Thereby, the integration pattern of vascular grafts and especially the graft (re-) vascularization are important factors to prevent infections as sufficient vascularization allows for transport of (immune) cells and antibodies of the innate and humoral immune system to the implantation site [50]. As a consequence, these cells and antibodies can eliminate infectious agents. Additionally, a suitable vascularization allows that administered antibiotics as treatment of vascular graft infections are also properly diffused within the implantation bed [31].

Based on these requirements, a novel xenogeneic vascular graft material won from porcine aorta was analyzed in the present study, while an already commercially available collagen-based vessel graft (XenoSure^®^, LeMaitre Vascular, Vaughan, ON, Canada) based on bovine pericardium was used as control material. Initially, the (ultra-) structure and the purification effort of the novel replacement material were examined based on previously described histological methods [38]. Furthermore, an established subcutaneous implantation model in Wistar rats was used to analyze the biocompatibility, the integration behavior, and the immune response of the novel graft material.

The analysis of the (ultra-) structure of the native pericardium-based vascular patch (BPP) showed that the material seems to be single-layered, which might represent the former pericardium fibrosa [51]. This layer is a compact structure composed of woven collagen bundles that are diversely distributed, which seems to be the basis of this medical device [51]. Moreover, the results showed that the BPP contained clearly visible remaining donor cells or cell remnants and cellular debris, which have manifoldly been suspected of leading to implant calcification or rejection, both being reasons for implant failures [51,52]. In this context, it has been stated that both xenogeneic and allogeneic cellular antigens are recognized as foreign by the host organism and therefore induce an inflammatory response or an immune-mediated rejection of the tissue [53]. In contrast, components of the extracellular matrix (ECM) are generally conserved among species and are tolerated well [53]. It must be mentioned that the overall goal of every decellularization process is to achieve the highest possible degree of preservation of the nativity of the ECM while at the same time removing as completely as possible immunologically effective components such as cells. Cellular remnants have already been described to be contained in different medical devices that are also already commercially available and that are clinically applied in daily practice [54,55]. Interestingly, it has manifoldly been shown that also the application of these materials leads to satisfying clinical results [54,55]. Thus, the question arises if the observed reaming cells or cell remnants will lead to undesired clinical outcomes. It is additionally questionable which decellularization protocol can lead to the deactivation of the epitopes that are responsible for triggering immunological cascades that will finally result in implant failures. Even this topic should be examined more deeply as these results are fundamental for almost all medical areas that apply collagen-based biomaterials.

In the case of the porcine aortic patch (PAP), it was shown that this novel biomaterial maintained its native structure of the origin tissue. Thus, it has been shown to be composed of three tunicae of the aortic wall after decellularization. Initially, the ECM part of the innermost layer of the aorta, the tunica intima, which is physiologically consisting of a thin layer of elastin and endothelial cells, was observable. Furthermore, the middle and most prominent layer, the tunica media, which consists of smooth muscle cells interposed with elastic fibers and loose collagen layers, has been clearly found. Finally, the outermost layer, the tunica adventitia, which represents a loose connective tissue including the vasa vasorum as tissue-specific vascularization of the aorta and its nerve tissue, has been identified. In contrast to the above-described BPP, no cells or cell remnants were found in any of the layers, while the structure and, thus, the nativity of the donor tissue seems to be preserved. This means that no foreign body responses or material-associated rejection based on immunological interventions with remaining cellular epitopes are to be expected.

Another interesting topic even in this context is the mechanical properties of vascular graft material. Although this characteristic was not directly examined in the present study, the analysis of the (ultra-) structure of both grafts can serve as a first indicator. Interestingly, both patches are won from precursor tissues from the vasculature (PAP) or related to the vasculature (BPP), and it can be concluded that the ECM properties are oriented to provide the desired mechanical properties need for their application as vascular patches. While the ECM of the pericardium in the case of the BPP has to withstand (permanent) mechanical stress, it has moreover been described that its fixation with glutaraldehyde enhances additionally its mechanical properties [56]. Thus, its suitability in terms of mechanical properties seems to be sufficient, especially since its successful application has already been described [56,57]. Considering that the PAP maintained its anatomic structure after decellularization, it can possibly indicate that the mechanical properties of the porcine aorta are also preserved. In this context, Beaufort et al. compared the mechanical properties of human and porcine aortas and found them to be comparable [58]. Hence, the use of PAP in vascular reconstruction can be advantageous in terms of mechanical matching that prevents high stresses on anastomotic suture sites and the development of intimal hyperplasia [45]. In turn, this would lower the incidence of stenosis and/or pseudoaneurysm as described in the case of the BPP [43,45]. However, this analysis was not part of the present study and should thus be analyzed in further material examinations.

The histopathological analysis showed that both vascular grafts exhibit different integration patterns. The tissue response to the BPP was similar at both material surfaces reflecting the single-layer character of the biomaterial. At day 10 post implantationem, an inflammatory tissue reaction based on mononuclear cells was observed, while a tissue response mainly based on macrophages and multinucleated giant cells (MNGCs) was detected at both material surfaces up to day 90 post implantationem. At day 120 post implantationem, a “passivation” of the tissue reaction was detectable as mainly macrophages were found at the material interfaces. During the study, only the superficial material regions were migrated with cells, while the inner cores were free of cellular invasion. Interestingly, a low migration of both macrophages and fibroblasts was observable at day 90 post implantationem. Altogether, this integration behavior leads to the conclusion that the material undergoes a long-term integration serving as a scaffold for delayed cellular remigration. Altogether, no exaggerated tissue responses were found related to this biomaterial, which underlines previous study results [59]. Thereby, the observed inflammatory tissue response may be related to the fixation in a sterile phosphate-buffered saline solution containing 0.2% glutaraldehyde as especially the latter is known to induce collagen crosslinking and thus inflammatory tissue reactions [60]. However, the observed tissue reaction is not exaggerated, and a sufficient standing time combined with a late cellular migration into the patch was observed. Interestingly, the manufacturer states that application of the “glutaraldehyde-fixed bovine pericardium may be subject to late attack by the immune system with subsequent tissue deterioration” [61]. However, this phenomenon was non-observed in the present study, which leads to the conclusion that the application of this patch is suitable for the proposed clinical indications. However, the observation period of the present study might be too short, so that further studies should also focus on later study time points, also up to the complete degradation of the biomaterial.

Interestingly, the PAP material induced three different tissue responses related to the above-described three layers. The former tunica intima induced a low-grade tissue response, including mainly macrophages and only sporadic MNGCs up to day 90 post implantationem, while passivation was also found at day 120 post implantationem related to this material part comparably to the BPP group. More contrary, the former tunica adventitia induced a stronger inflammatory tissue response, including higher numbers of macrophages and MNGCs up to day 60 post implantationem in concert with a higher related vascularization pattern even in this implant bed region. Furthermore, it was shown that this material part was degraded at day 90 post implantationem leading to a “passivated” tissue rection up to the end of the observation period. Moreover, the middle layer, the former tunica media, showed a stepwise cellular remigration starting with day 60 up to day 120 post implantationem. In contrast to the integration behavior of the BPP material, which was invaded by single cells, a cell invasion of mononuclear cells was observed at 60 post implantationem that was replaced by a more complex tissue ingrowth starting with day 90 post implantationem including mainly macrophages and fibroblasts beside single multinucleated giant cells and blood vessels. However, no breakdown or material fragmentation has been observed up to day 120 post implantationem in the case of this material layer.

These results lead to the conclusion that the tissue reactions related to both the former tunica intima and tunica media were generally comparable to that also found in the PAP group. Thus, it could also be concluded that especially the material part of the tunica media might undergo a long-term integration serving as a scaffold for stepwise cellular remigration. Even the observation of the migration of macrophages and fibroblasts in both study groups might hint at a tissue regeneration process as it was shown that both cell types can be transformed into myofibroblasts that allow regeneration of the contractile part of the vessel wall [62,63]. Moreover, the former tunica media of the PAP graft was well vascularized even at the end of the observation period. This integration behavior might, on the one hand, additionally support tissue regeneration due to the transport of nutrients and, more important, the migration of precursor cells that might then migrate to the defect area [31].

Moreover, the integration behavior of the former tunica adventitia is remarkably interesting even in the context of tissue regeneration up to the condition of a restitutio ad integrum. This layer is chiefly made of loose collagen, which possibly was the reason behind its much faster degradation compared to the other components of the PAP. The tunica adventitia was completely resorbed by day 30; however, its site appeared to be highly vascularized. This observation is significant in terms of the PAP having a biomimetic vascularization pattern compared to its physiological vasa vasorum, as well as in jumpstarting the vascularization from an early time point post implantationem.

In this context, the occurrence of higher numbers of MNGCs within this implant part is of special interest. The location of the MNGCs can be of significance as their correlation with higher vascularization has still been shown in the case of different other biomaterials such as bone substitute materials [64]. Thereby, this cell type, together with other cells such as macrophages, are prominent inductors of the process of angiogenesis based on their expression of the vascular endothelial growth factor (VEGF) [65,66,67].

Moreover, the higher early and late vascularization pattern of the PAP graft is of the highest interest in the context of graft infections. Thus, it is thinkable that the higher vascularization followed by increased perfusion of the implantation site will allow for better transport of intravenous antibiotics to the site of infection to prevent and/or fight infections. Specifically, because trans-vascularization of the implants was well developed after 4 months, the same time period after which latent or delayed infections start to manifest. As aforementioned, current treatment regimes for the higher classifications include surgical intervention for debridement or, in most cases, excision of the graft and reimplantation of new material. In contrast, no new vessels were spotted within the core of BPP implants, and the vascularization remained on the peripheries, and at later time points, vascularization appeared to slowly migrate toward the body of the implant, which might restrict the application of this biomaterial in many cases, even in favor of the novel graft material. However, this implantation model cannot precisely predict the vascularization pattern of both vascular grafts, so that further studies with implantation at the “target site” must prove the functionality or effectiveness of the new graft material.

Additionally, the presence of a functional vasculature also allows the recruitment of reparative cell types such as circulating progenitor cells or reparative myeloid cells, which can contribute to tissue regeneration and remodeling [68]. Therefore, a rapid (re-) vascularization within some days after material application is of vital importance for the function of such graft materials and, thus, of great clinical relevance [69].

Finally, the histomorphometrical analysis showed that the percentages of anti-inflammatory M2 macrophages appear comparable for both the BPP and PAP implants over the complete study period. However, initial and delayed M1 percentages were significantly higher in BPP implants compared to PAP implants. Moreover, the M1 concentrations in BPP implants were partially significantly elevated compared to the M2 phenotype. This insight suggests that BPP implants healing process can possibly be inhibited or hindered by the ongoing and increasing activation of M1 macrophages. In contrast, M1 and M2 macrophages maintained balanced and comparable percentages throughout all time points in the group of the PAP implant, suggesting integrative tissue healing patterns.

In this context, it has already been revealed that both phenotypes of macrophages, M1 (pro-inflammatory) and M2 (anti-inflammatory), have been shown important determinants for the process of tissue regeneration [70]. For instance, their activation in concert with each other allows for simultaneous tissue remodeling and increased vascularization [66,70,71]. Lee et al. described the polarization of macrophages into M1 phenotypes as a typical reaction to foreign bodies; however, their persistence for elongated times is associated with poor tissue regeneration [71]. In contrast, M2 macrophages have manifoldly been correlated with the promotion of tissue repair by underlying promotion cascades such as are known to promote tissue-repair functions such as wound debridement, cell proliferation, angiogenesis, and matrix remodeling [71].

Another very interesting finding of the present study is the phenotypic alignment of the material-associated MNGCs that only stained positive to the M1 marker, which suggests its phagocytotic activity, comparable to their involvement in the degradation of different other biomaterials [38,72]. It is worth mentioning that M1 MNGCs were seen at areas where new vessels were also detected, for instance, within the tunica adventitia of the PAP implants. This observation verifies the angiogenetic capacity of the phagocytosing M1 macrophages and MNGCs. Altogether, these findings are valuable as macrophage infiltration has also been inversely correlated with the inhibition of stenosis [73]. This means that an escalated foreign body response can predict a higher incidence of stenosis; however, both vascular graft implants induced acceptable tissue responses and can be classified as biocompatible.

Altogether, the study results show that the novel porcine aortic vascular grafts serve as a xenogeneic alternative that can potentially overcome complications that usually accompany the most and commonly used vascular xenografts, the bovine pericardial patches used as control material in the present study. PAP implants seemed to be advantageous in many aspects: (i) cellular infiltration and host tissue integration, (ii) vascularization pattern that branches to all parts of the graft, and (iii) balanced immune tissue reaction that can result in less scar tissue and enhanced integrative healing patterns. Early infection of the vascular implant depends on many factors that are influenced by the handling physician, operative time, and the implantation site [30]. For instance, inguinal vascular reconstruction has been reported with higher infection rates than those in thoracic and abdominal vascular reconstructions [30]. However, late infection, due to bacterial deposition or dormant bacterial activation, largely depends on the type of vascular graft and its development in vivo. Therefore, the unique trans-implant vascularization can provide unprecedented anti-infection properties that can avoid the production of antibiotic-resistant biofilms. In addition, initial results foresee better mechanical matching that is key to implant success and durability. Additionally, further in vivo studies have to prove the mode of action of the novel vessel graft as the validity of the present study might be restricted. Thereby, it must be analyzed if the integration behavior after its proposed application is comparable to that won by the subcutaneous implantation model.

## 4. Materials and Methods

### 4.1. Decellularization of Native Porcine Aorta

Porcine aortas were excised from full-grown swine during slaughter. The upper layer of the adventitia was carefully manually removed, and the prepared aortas were stored into a 0.1 M ethylenediaminetetraacetic acid (EDTA; Carl Roth, Karlsruhe, Germany) solution.

The decellularization process included incubation steps in diverse chemicals as follows: the samples were initially washed in demineralized water for 60 min and then incubated in sodium hypochlorite solution (0.5%) (Bioanalytic GmbH, Freiburg, Germany) for 10 min. Afterward, an incubation in TSP buffer (pH 7.4; PanReac AppliChem ITW Reagents, Darmstadt, Germany) was performed for 60 min, followed by treatment with hydrogen peroxide (TH. Geyer, Renningen, Germany) for 60 min. The next incubation step included incubation in 0.02 M phosphoric acid (PanReac AppliChem ITW Reagents, Darmstadt, Germany) for 19 h. Finally, the samples were transferred in TSP buffer (pH 7.4) and incubated for 3 h. Decellularized samples were kept in a 0.1 M EDTA and 3% sodium chloride (Carl Roth, Karlsruhe, Germany) at 4 °C.

### 4.2. Xenosure^®^ Biologic Patch

Xenosure^®^ (XenoSure^®^, LeMaitre Vascular, Vaughan, ON, Canada) is an incised piece of bovine pericardium tissue. The patch is decellularized with chemical processes, and as a post-purification process, the patch is treated with glutaraldehyde for further purification and crosslinking. Indications for this patch include vascular/cardiac repair, soft tissue reconstruction, suture reinforcement, and duraplasty.

### 4.3. In Vivo Study

The in vivo experiments were executed in cooperation with the Faculty of Medicine (University of Niš, Serbia) after initial approval by the Local Ethical Committee on the basis of the Veterinary Directorate of the Ministry of Agriculture, Forestry and Water Management of the Republic of Serbia issued the decision number 323-07-00278/2017-05/6 (Date: 13 July 2017). Thereby, the experimental animals were housed using standard conditions, i.e., water ad libitum, artificial light, and regular rat pellet, combined, and standard pre- and postoperative care were conducted.

In total, 50 females, 6–8-week-old Wistar rats obtained from the Military Medical Academy (Belgrade, Serbia) were split into two study groups, each containing 25 experimental animals and five animals per time point (*n* = 5) for 10, 30, 60, 90, and 120 days. An established and standardized subcutaneous implantation model was used following the DIN ISO 10993-6:2016 guidelines [74,75].

Briefly, the rostral subscapular regions of the experimental animals were shaved and disinfected after initial anesthesia by means of an intraperitoneal injection (10 mL ketamine (50 mg/mL) with 1.6 mL Xylazine (2%)). Afterward, an incision was made, followed by blunt generation of a subcutaneous pocket and insertion of the biomaterials. Finally, the operation wounds were stitched.

At different time points, the animals were euthanized by means of an anesthesia medication overdose. Directly afterward, the biomaterials and the neighbored tissue were excised, subsequently fixed with a 4% formalin solution for 24 h, and histologically processed as specified below. Initial dehydration via a series of increasing alcohol concentrations ending up with xylol exposure and paraffin embedding was performed. This procedure allowed to produce tissue sections with a thickness of 3–5 μm using a rotation microtome (SLEE, Mainz, Germany). Hematoxylin and eosin (H&E), Movat pentachrome, and alcian blue staining were conducted for every explant. Additionally, immunohistochemical stainings of the M1 and M2 subforms of implant bed macrophages by means of antibodies against the pro- and anti-inflammatory molecules hemoglobin scavenger receptor (CD163) (CD163) (anti-CD163-antibody, ab182422, dilution: 1:500, abcam, Cambridge, UK) and CD11c, also known as Integrin alpha X (anti-CD11c-antibody, abx231412, dilution 1:200, Abexxa Ltd., Cambridge, UK) based on previously published methods were prepared [35]. Furthermore, immunohistochemical stainings of the blood vessels within the implant beds by means of antibodies against CD31 (anti-CD31-antibody, ab182981, dilution 1:2000, abcam, Cambridge, UK) was prepared, as published previously [35,65].

In brief, initial treatment of the slides using citrate buffer and proteinase K at pH8 for 20 min in a water bath at 96 °C was performed, and afterward, equilibration via TBS-T buffer was conducted. Then, the tissue slides were treated with H_2_O_2_ and avidin and biotin blocking solutions (Avidin/Biotin Blocking Kit, Vector Laboratories, Burlingame, CA, USA) followed by incubation with the respective above-mentioned antibody for 30 min. This step was directly followed by incubation with the secondary antibody (goat anti-rabbit IgG-B, sc-2040, 1:200, Santa Cruz Biotechnology, Shandon, CA, USA) and the avidin–biotin–peroxidase complex (Thermo Fisher Scientific, Dreeich, Germany) (30 min). For finalization, counterstaining by hematoxylin and blueing was applied.

**Figure 9:**
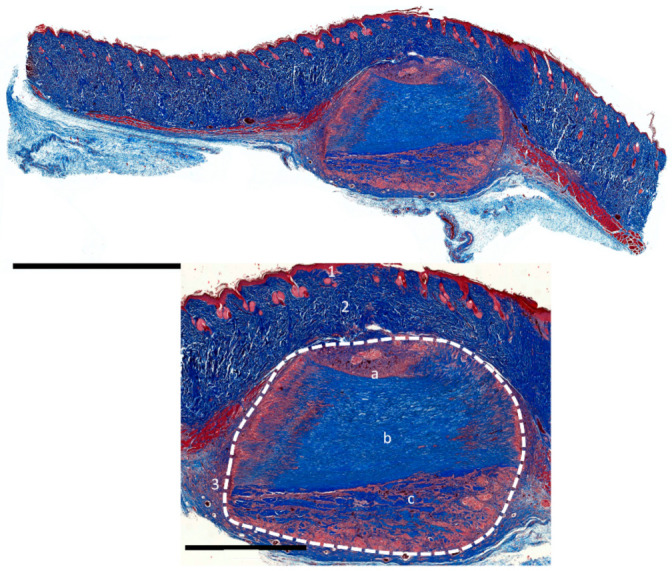
Cross-sectional overview and closeups of the subcutaneous implantation model of the porcine aortal patch (PAP). Dashed line: implant, 1: epidermis, 2: dermis, 3: subcutaneous layer, a: tunica intima, b: tunica media, and c: tunica adventitia. (Images were stained with Azan, magnification: 20× and scale bars = 5 mm (overview) and 2 mm (magnified)).

These stainings were used for histopathological analyses of the tissue–biomaterial interactions by means of an Axio.Scope. A1 microscope (Zeiss, Oberkochen, Germany) based on a standardized protocol [55,64,65,76,77]. Thereby, the focus was on the evaluation of the following parameters: fibrosis, hemorrhage, necrosis, vascularization, and the presence of neutrophils, lymphocytes, plasma cells, macrophages, and biomaterial-associated multinucleated giant cells (BMGCs). Furthermore, the integration behavior of the biomaterials was comparatively analyzed. Microphotographs were made using an Axiocam 305 color and the ZEN Core software (Zeiss, Oberkochen, Germany). Figure 9 shows the subcutaneous implantation model with the specific orientation of the aortal patch. The most inner tunica of the aorta (tunica intima) was the closest to the dermis, and the most outer tunica was the furthest (tunica adventitia).

Additionally, the immunohistochemically stained slides were used for histomorphometrical measurements of the occurrence of macrophage subtypes within the implant beds of the vascular graft materials as previously published [55,64,65,76,77]. Briefly, “total scans” were assembled by means of a scanning microscope composed of an Axio Scope.A1 light microscope, an Axiocam 305 color digital camera, and an automatic scanning table (Maerzhaeuser, Wetzlar, Germany) in combination with the ZEN Core software (all: Zeiss, Oberkochen, Germany). Then, the cells were counted using a specialized ImageJ plugin as previously described [39].

### 4.4. Statistical Analysis

The study data were graphed as mean and standard deviation following an analysis of variance (ANOVA) by means of the GraphPad Prism 8.4 software (GraphPad Software Inc., La Jolla, CA, USA). Statistical differences were stated as significant if the p-values were less than 0.05 (* *p* ≤ 0.05) and highly significant if the p-values were less than 0.01 (** *p* ≤ 0.01) or less than 0.001 (*** *p* ≤ 0.001).

## Figures and Tables

**Table 1:** Histomorphometrical results of the percentages of pro- and anti-inflammatory cells in the implant beds of the porcine aorta patch (PAP) and the bovine pericardial patch (BPP).

Patch/Time Point	Day 10	Day 30	Day 60	Day 90	Day 120
**CD163**					
**PAP**	3.00% ± 2.23%	4.57% ± 1.86%	2.65% ± 1.03%	2.63% ± 0.94%	3.45% ± 1.20%
**BPP**	1.96% ± 1.17%	1.6% ± 1.33%	1.68% ± 0.88%	2.04% ± 0.59%	2.21% ± 0.59%
**CD11c**					
**PAP**	2.53% ± 1.20%	3.47% ± 1.38%	5.69% ± 0.85%	4.31% ± 1.46%	3.72% ± 0.40%
**BPP**	7.85% ± 1.34%	3.90% ± 1.59%	6.58% ± 2.65%	5.61% ± 2.11%	9.85% ± 1.71%

**Table 2:** Classification of vascular graft infections [49].

Classification	Indications
I	Infection of the surrounding dermis.
II	Infection of the surrounding subcutaneous tissue.
III	Infection of the body of the graft.
IV	Infection of the body of the graft and the anastomosis.
V	Infection of the body of the graft and the anastomosis + bacteremia and/or bleeding of anastomosis.

## Data Availability

Data is contained within the article.
